# Can exhaled NO fraction predict radiotherapy-induced lung toxicity in lung cancer patients?

**DOI:** 10.1186/1748-717X-7-117

**Published:** 2012-07-28

**Authors:** Irina Enache, Georges Noel, M-Young Jeung, Nicolas Meyer, Monique Oswald-Mammosser, Emile Urban-Kraemer, Catherine Schumacher, Bernard Geny, Elisabeth Quoix, Anne Charloux

**Affiliations:** 1 Pôle de Pathologie Thoracique, Hôpitaux Universitaires, BP 426, 67091, Strasbourg Cedex, France; 2 Département Universitaire de Radiothérapie, Centre de lutte contre le cancer Paul Strauss, 3, rue de la Porte de l’Hôpital, BP 42, 67065, Strasbourg, France; 3 Service de Radiologie B, Hôpitaux Universitaires, BP 426, 67091, Strasbourg Cedex, France; 4 Laboratoire de Biostatistique, Université de Strasbourg, Faculté de Médecine, 4, rue Kirschleger, 67085, Strasbourg, France; 5 Service de Physiologie et d’Explorations Fonctionnelles, Pôle de Pathologie Thoracique, Hôpitaux Universitaires, BP 426, 67091, Strasbourg Cedex, France

**Keywords:** Chemotherapy, Radiotherapy, Radiation pneumonitis, Exhaled nitric oxide, Lung cancer

## Abstract

**Background:**

A large increase in nitric oxide fraction (FeNO) after radiotherapy (RT) for lung cancer may predict RT-induced lung toxicity.

**Methods:**

In this study, we assessed the relationships between FeNO variations and respiratory symptoms, CT scan changes or dose volume histogram (DVH) parameters after RT. We measured FeNO before RT, 4, 5, 6, 10 weeks, 4 and 7.5 months after RT in 65 lung cancer patients.

**Results:**

Eleven lung cancer patients (17%) complained of significant respiratory symptoms and 21 (31%) had radiation pneumonitis images in >1/3 of the irradiated lung after RT. Thirteen patients (20%) showed increases in FeNO >10 ppb. The sensitivity and specificity of a >10 ppb FeNO increase for the diagnosis of RT-associated respiratory symptoms were 18% and 83%, respectively. There was no correlation between DVH parameters or CT scan changes after RT and FeNO variations. Three patients (5%) showed intriguingly strong (2 or 3-fold, up to 55 ppb) and sustained increases in FeNO at 4 and 5 weeks, followed by significant respiratory symptoms and/or radiation-pneumonitis images.

**Conclusion:**

Serial FeNO measurements during RT had a low ability to identify lung cancer patients who developed symptoms or images of radiation pneumonitis. However, three patients presented with a particular pattern which deserves to be investigated.

## Background

Measurement of exhaled nitric oxide fraction (FeNO) is a non-invasive, well standardized, simple technique and is regarded as a potential tool for screening and follow-up of chest diseases. FeNO is increasingly used to monitor airway inflammation in patients with asthma [1]. It also may be useful in predicting steroid responsiveness in chronic obstructive pulmonary disease [2], evaluating response to medical therapy in pulmonary hypertension [1], and identifying lung function deterioration in scleroderma [3]. While the need of biomarkers for early detection and follow-up of chest malignancies has been recently highlighted [4,5], data on FeNO level at diagnosis or treatment-induced variations of FeNO are few. FeNO has been found to be increased in patients with Hodgkin disease, and to normalize after remission [6,7]. FeNO from lung cancer patients has also been found higher than that from controls [8,9], and associated with an up-regulation of inducible NO synthase (iNOS) activity in alveolar macrophages[8]. Serial measurements have demonstrated that FeNO levels diminished after chemotherapy [10] and/or radiotherapy (RT) [11].

Pulmonary toxicity remains a dose-limiting side-effect of RT. It can impair the quality of life of patients, and be fatal in few of them. Multi-parameters models based on dosimetric data and patients’ characteristics have been built to predict radiation-induced lung toxicity. However, efficiency of these models is limited, and efforts to identify additional prognostic factors, including biomarkers, are in progress [12,13]. Interestingly, in a series of 29 lung cancer patients, 5 demonstrated a three-fold increase in FeNO during RT, and 3 of them subsequently developed radiation pneumonitis requiring steroids [11]. In a study of oesophageal cancer, a 1.5-fold increase in FeNO was observed in 4 patients with radiation pneumonitis symptoms, but not in the 24 asymptomatic patients. Moreover, the interval to the occurrence of peak symptoms was inversely related to the increase in FeNO [14]. In another study by the same team, performed in 50 patients with lung and oesophageal cancer, all symptomatic patients had a >1.4-fold increase in FeNO at the end of RT [15]. These studies suggest that FeNO may be used for early detection of radiation-induced lung injury. To extend these results, we assessed the relationship between radiation-induced lung injury and FeNO variations in 65 lung cancer patients.

This study was designed i) to describe the time-course of FeNO till 7.5 months after 3D conformal RT ii) to assess the relationship between FeNO variations and dose-volume histogram analysis iii) to assess the relationship between FeNO increases (using recommended criteria [1]) and respiratory symptoms or CT scan changes after RT for lung cancer.

## Methods

### Patients

From October 2005 to February 2010, 65 patients with histologically or cytologically confirmed unresectable lung cancer accepted to participate and signed an informed consent prior to registration in this observational study, which has been approved by the Institutional Review Board (HUS n°3492). Lung cancer patients with stage I-III, with or without pre-RT surgery or chemotherapy were eligible. Patients were ineligible if they were younger than 18 years, had history of prior RT to the thorax, had an ECOG performance status >2, had active systemic or pulmonary infection, asthma, atopy, or synchronous malignancy within 2 years of entry.

### Radiotherapy modalities

Patients were immobilized in a supine position with arms above the head. Target volumes were delineated on planning CT scan with 2.5 mm slices. The gross tumour volume (GTV), clinical target volume (CTV), and planning target volume (PTV) were delineated according to the definitions of the International Commission on Radiation Units and Measurements [16], the GVT including tumour and nodes visualised on CT and positron emission tomography (PET) -CT scan. CTV was defined as the GTV with a 5-mm margin for the mediastinal areas and a 5–8 mm margin for the primary tumour according to the tumour histology. PTV (including set-up margin and internal target volume) was obtained by a 2–10 mm 3D-expansion from CTV. In case of induction chemotherapy, the post-chemotherapy volume of the primary tumour was considered as the GTV of the primary tumour and the pathological lymph node areas positive on the pre-treatment PET scan were included in the GTV. After surgery, the CTV included the scar or residual tumour, the pathological lymph node areas and lymph node areas just above and below the pathological one. Irradiation was applied with 2 to four 6–25 MV photon beams from accelerators with multileaf linear collimators. Portal imaging for set-up control was performed daily. The irradiation dose was delivered with conventional fractionation (2 Gy per fraction, 5 fractions per week) up to 66 Gy. After lung resection, the dose was 54 Gy for R0 resection and no nodal capsular rupture. If resection was not R0, or nodal capsular rupture was diagnosed, the dose was increased up to 66 Gy.

#### Dose Volume Histogram (DVH) parameters

Doses were calculated to account for tissue density heterogeneity. The lungs were automatically contoured, excluding the GTV. Mean lung dose (MLD, average dose to the CT-defined total lung volume, excluding the GTV), V5, V13, V20 and V30 (percentage of lung volume receiving at least 5, 13, 20 or 30 Gy) were calculated from the lung DVH.

#### Lung toxicity

Post-treatment cough or dyspnea grade 2 or higher according to Common Terminology Criteria of Adverse Events (CTCAE) v3.0, or significant increases in symptoms if patients presented with pre-existing symptoms, were regarded as significant lung toxicity. These patients were categorized as “symptomatic patients”.

### CT Scan changes after radiotherapy

CT scan were performed within 4 weeks before, and 10 weeks, 4 and 7.5 months after the beginning of RT and reviewed at the same time for each patient by the same radiologist experienced in CT of the thorax. The system used to score the extent of emphysema on the CT scans was adapted from prior work by Goddard et al. [17] and Bankier et al. [18]. Each CT section was assessed individually, and the right and left lungs were graded separately according to the percentage area that demonstrated changes suggestive of radiation pneumonitis. These changes were ground-glass opacity, consolidation, fibrosis, traction bronchiectasis, scarring, and volume loss. Radiation-induced manifestations were scored in the right lung and in the left lung independently, as follow:

0 : none

<1 : minimal radiographic findings, volume <1 cm^2^

1 : changes with estimated proportion of lung volume of <1/3

2 : changes with estimated proportion of lung volume of >1/3 and <2/3

3 : changes with estimated proportion of lung volume of >2/3

Scores obtained in the right and in the left lung were then summed up to provide the grade for the whole lung. This yielded maximum possible scores of 3 for the left side and 3 for the right side, for a maximum possible total score of 6. In this study, we regarded scores ≥2 as significant.

### Exhaled NO measurement

Fractional exhaled NO was measured according to the ATS–ERS guidelines [19]. In brief, FeNO was measured online with a chemiluminescence analyser (NIOX; Aerocrine, Solna, Sweden). The patients were instructed to inhale to near-total lung capacity and to exhale immediately at a constant flow rate of 50 ml/s during 10 sec. FeNO was calculated during the last 3 seconds of the exhalation. Measurements were performed in triplicate to obtain three acceptable FeNO measurements out of a maximum of six attempts. Exhalations were approved if they did not deviate more than 10%. FeNO was expressed as the average of these three measurements. With the NIOX system, a FeNO variation >4 ppb is regarded as significant [20]. Some patients accepted to perform FeNO measurements at various exhalation flow rates, i.e. 30, 50, 80 and 150 ml/s. Duration of exhalation ranged from 12 sec for 30 ml/s to 8 s for 150 ml/s. For each flow rate we used the mean value of two acceptable measurements. Elimination rates of NO during each exhalation were plotted against exhalation flow rates using a linear regression model. The slope of this regression line represents the alveolar NO concentration (Calv, ppb), and the intercept represents the bronchial NO flux (JNO, pl/s) [21]. Alveolar and bronchial NO values were determined when the correlation coefficient of the regression model was at least 0.9.

Exhaled NO measurements were performed within 2 weeks before RT, and 4, 5, 6, 10 weeks, 4 months and 7.5 months after the beginning of RT. Smoking and treatment with inhaled or oral corticosteroids were noted before each measurement.

### Statistical analysis

Results are expressed as mean (standard deviation) or proportions. Characteristics of the patients were compared using *t*-test or chisquare test depending on the variable being continuous or qualitative. Changes in FeNO with time were evaluated using linear mixed models with unstructured variance-covariances matrices. Sensibility, specificity, positive and negative predictive values of FeNO changes to predict respiratory symptoms grade ≥2 or CT-scan images score ≥2 during the 7.5 months following RT were calculated with cut-off values of 10 ppb, 4 ppb, and 2 fold-increase. According to the American Thoracic Society criteria [1], in patients, an increase in FeNO higher than 10 ppb for FeNO values lower than 50 ppb from one visit to the next is regarded as significant. Correlation at each time between FeNO and DVH parameters or score of CT-scan image was computed using Pearson or Spearman correlation coefficient. The significance level was set at alpha = 0.05. Computations were done with SAS 8.0 (PROX MIXED) and R 2.11.

## Results

### Patients’ characteristics, treatment and RT-induced symptoms

Patients’ characteristics are reported in Table 1. All patients received the planned RT dose. Disease progression was diagnosed in seventeen lung cancer patients within the study period. Eleven lung cancer patients (17%) developed or increased significantly respiratory symptoms, 4 during RT, 2 during the 3^rd^ month, 5 during the last month of the study, and were diagnosed with radiation pneumonitis.

**Table 1:** Patients’ characteristics

	**Lung cancer patients (n = 65)**
**Males/Females**	48/17
**Age** (years, mean (SD))	61 (11)
**Smoking history**	Current smoker 13
	Ex smoker 49
	Never smoker 3
**Histology and stage**	**NSCLC**
	squamous : 32
	adenocarcinoma : 12
	large cell : 4
	unspecified : 4
	Stage : I : 1
	II : 4
	III : 47
	**SCLC** : 13
	Stage : limited:
**Treatment**	sequential chemo-RT : 21
	concurrent chemo-RT : 40
	no chemotherapy : 4
	lung resection before RT : 14
**DVH (mean (SD))**	V5: 45% (15%)
	V13: 31% (12%)
	V20: 24% (10%)
	V30: 17% (8%)
	MLD: 12.9 Gy (4.6 Gy)
**Baseline PFT**	Normal values : 15 Restrictive ventilatory defect : 13
	Obstructive ventilatory defect : 37

(N)SCLC: (non) small cell lung cancer. DVH: dose volume histogram, MLD: mean lung dose (Gy), PFT: pulmonary function test, V5, 13, 20, 300: percentage of lung volume receiving at least 5, 13, 20 or 30 Gy (%).

### CT scan changes after radiotherapy

Results are reported in Table 2. Twenty-one (31%) of lung cancer patients developed images score ≥2, at 10 weeks, 4 or 7.5 months.

**Table 2:** CT-scan image scores after radiotherapy

**Score**	**10 weeks**	**4 months**	**7.5 months**
	***n = 50***	***n = 53***	***n = 49***
0- <1	36 (72%)	22 (42%)	18 (37%)
1-1.5	9 (18%)	17 (32%)	19 (39%)
2-2.5	3 (6%)	9 (17%)	9 (18%)
3 - >3	2 (4%)	5 (9%)	3 (6%)

### Time course of FeNO and significant FeNO variations

**Figure 1:**
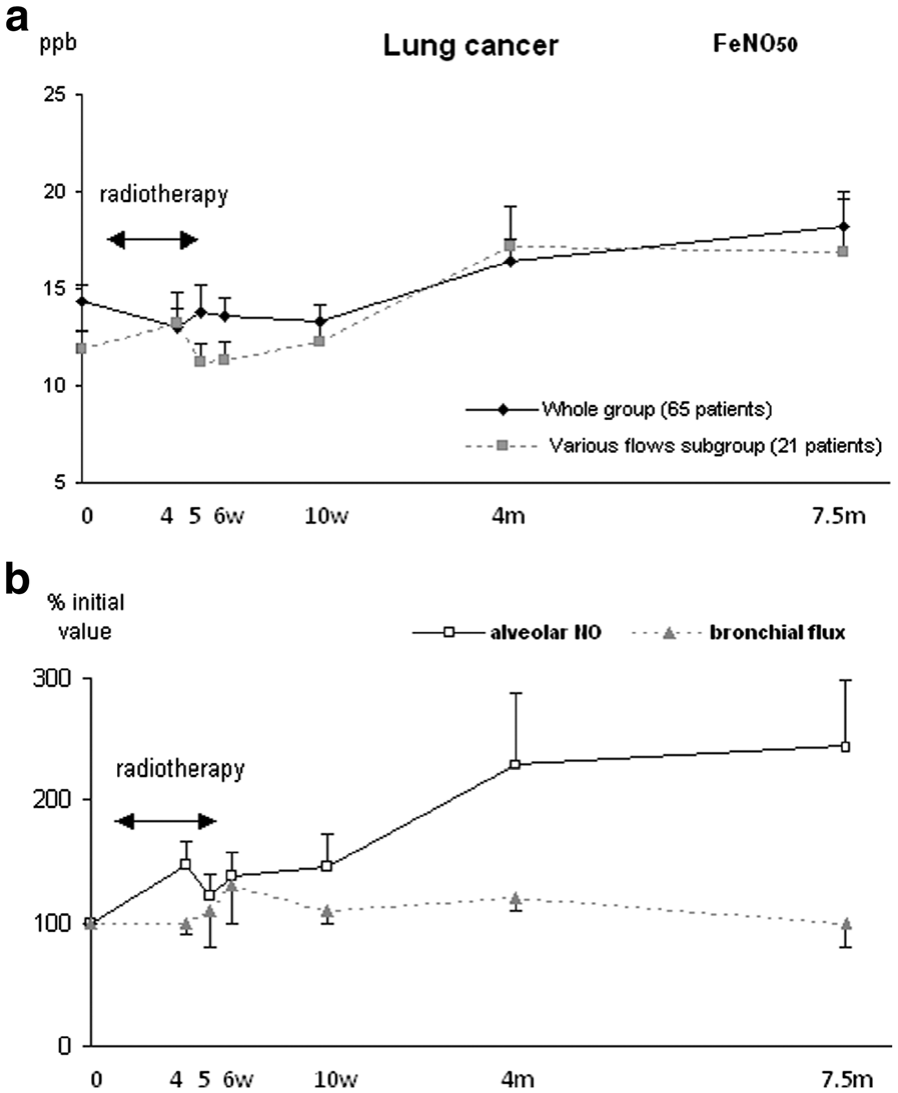
**Time course of FeNO (a), alveolar NO and bronchial NO flux (b) in lung cancer patients (mean (SEM)).** (**a**) FeNO measured at 50 ml/s (FeNO_50_) in the whole group (solid line, 65 patients), and in the subgroup who accepted to perform measurements at various exhalation flows to calculate alveolar NO and bronchial NO flux (dotted line, 21 patients). See text for comments.

Before RT, FeNO was in the range of normal values (14.3 ppb (7.2)). A linear model fitted with the FeNO time trend (slope: 0.15, p < 0.001). Mean FeNO increased from 14.3 (7.2) ppb before RT to 18.2 (12.5) ppb (+ 27% (8%)) at 7.5 months (Figure 1). Similarly, a linear model fitted with the time trend of alveolar NO (slope: 0.02, p < 0.01), which was higher at 7.5 months than before RT (0.24 (0.16) to 0.34 (0.16) pl/s, + 42% (6%)). Bronchial NO flux did not vary significantly after RT. These serial alveolar and bronchial NO measurements were obtained in 21 patients who accepted and succeeded in performing multiple exhalations at various flow rates.

Within the 10 first weeks period, 13 patients (20%) showed at least one increase in FeNO >10 ppb (9 patients showed one, 2 patients showed 2, and 2 patients showed 3 increases > 10 ppb).

### Relationships between FeNO and, CT scan, dosimetric parameters, or respiratory symptoms

**Figure 2:**
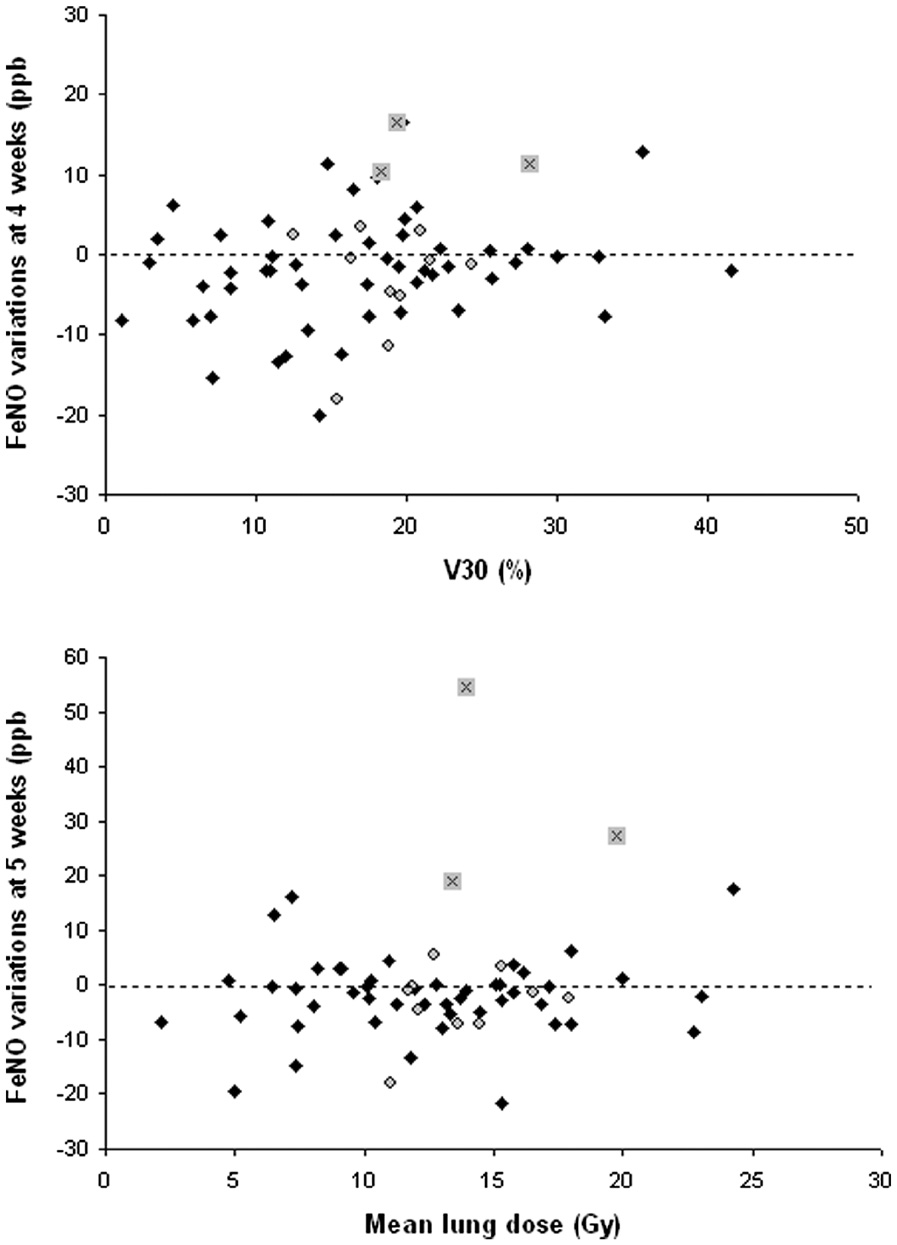
**Examples of relationships between dose volume histogram (DVH) parameters and FeNO variations in lung cancer patients at different time points.** Diamonds: asymptomatic patients. Circles: symptomatic patients. Black crosses: three patients with particular pattern (both strong and sustained FeNO increase: cf text for details).

**Figure 3:**
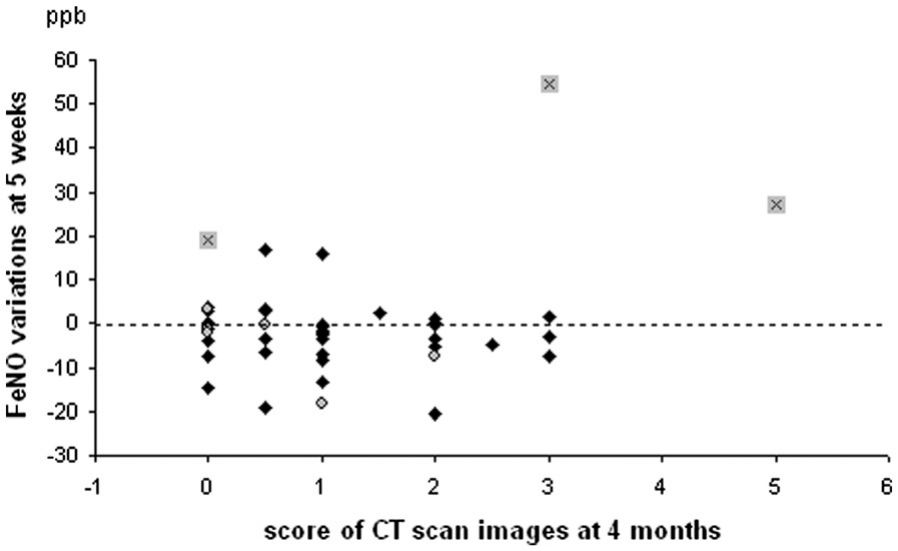
**Example of relationship between score of radiation-induced CT scan images and FeNO variations in lung cancer patients.** Diamonds: asymptomatic patients. Circles : symptomatic patients. Black crosses: three patients with particular pattern (both strong and sustained FeNO increase : cf text for details).

There was no correlation between DVH parameters and changes in FeNO (Figure 2). There was no correlation between absolute values or variations in FeNO and the score of CT scan changes after RT (Figure 3). However, all DVH parameters correlated with the score of radiation-induced CT-scan images at 4 months (with r from 0.31 to 0.41, p < 0.05) and 7.5 months (with r from 0.45 to 0.54, p < 0.001).

In symptomatic patients, mean FeNO values were 13.7 ppb (10.5) at 4 weeks, 17.7 ppb (21.5) at 5 weeks and 11.8 ppb (7.9) at 6 weeks. These values did not differ from those observed in asymptomatic patients, which were 13.0 ppb (6.4) at 4 weeks, 12.9 ppb (7.4) at 5 weeks and 14.0 (7.2) at 6 weeks.

The sensitivity of FeNO variations for the diagnosis of RT-induced symptoms grade ≥2 was low (18%) for a 10 ppb increase (at least one increase within the 10 first weeks). Specificity reached 83% (Table 3). The sensitivity and specificity to detect symptoms and/or CT-san images of score ≥2 were a little higher, reaching 22% and 87%, respectively. Using a >4 ppb increase (regarded as the lowest clinical significant variation with our device [20]) as a threshold for symptoms detection, offered a sensitivity of 36% and a specificity of 67%. 

**Table 3:** **Ability of a >10 ppb increase in FeNO**[1]**to predict respiratory symptoms (grade ≥2) and/or radiation-induced CT-scan images (score ≥2) after radiotherapy in 65 patients with lung cancer**

**>10 ppb increase in FeNO**	**Sensitivity**	**Specificity**	**Positive predictive value**	**Negative predictive value**
Respiratory symptoms	18%	83%	18%	83%
CT-scan images	24%	86%	45%	70%
Respiratory symptoms and/or CT-scan images	22%	87%	55%	61%

Eleven patients developed respiratory symptoms (17%). Thirteen (20%) showed at least one increase >10 ppb within 10 weeks.

**Figure 4:**
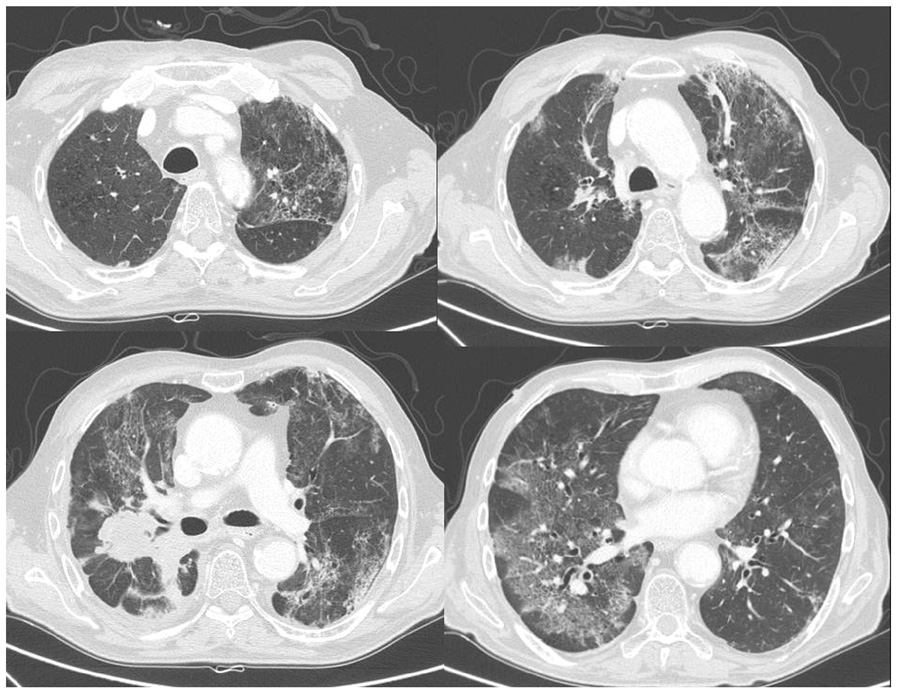
CT scan images observed 4 months after the beginning of radiotherapy, in one of the three patients with a strong and sustained increase in FeNO measured during radiotherapy.

Three patients showed a particular pattern, with very large and sustained rises of FeNO as early as 4 weeks: two symptomatic patients showed a 2-fold (up to +17 ppb) and 3-fold (up to +55 ppb) increase in FeNO, respectively; one patient regarded as free of radiation-induced symptoms (with pre-RT grade 1 cough, dyspnea, and sputum, which increased only slightly within 4 months) presented a 3-fold increase in FeNO (up to +27 ppb). These three patients had MLD of 13, 14, and 19 Gy, and presented with CT scan images score 1 at 10 weeks and 7.5 months (4 months : non available), score 3 at 4 months, and score 5 at 4 months, respectively (Figure 4). The mean FeNO values of these three patients were 27.2 ppb (8.0) at 4 weeks, 46.2 ppb (26.5) at 5 weeks and 26.2 ppb (5.8) at 6 weeks. This was significantly higher than the values observed in the other 62 patients (12.3 ppb (6.4) at 4 weeks (p < 0.05), 12.0 ppb (7.0) at 5 weeks (p < 0.01), and 12.9 ppb (6.8) at 6 weeks (p < 0.05)). Taking into account these patients, we evaluated the ability of FeNO to predict respiratory symptoms after RT with a 2-fold increase threshold (together with a significant increase of >4 ppb). Sensitivity was 18%, specificity, 98%, positive predictive value, 75% and negative predictive value, 85%.

### Corticosteroids or smoking and FeNO

During the study period, 9 patients were treated with inhaled corticosteroids (for chronic obstructive pulmonary disease) and 13 with oral corticosteroids (11 for radiation pneumonitis and 2 because of prophylactic brain irradiation performed during the last two months). The mean value of FeNO in treated patients was 12.8 (10.0) ppb, lower than in non-treated patients (15.5 (10.6) ppb, p < 0.05)). Thirteen patients continued smoking. The mean FeNO value was 13.2 (11.4) ppb in smokers, lower than in non-smokers (15.3 (8.8) ppb; p < 0.05). The increase in FeNO observed in lung cancer at 4 and 7.5 months was not due to patients who stopped smoking or had previous corticosteroids treatment. Among the 4 patients who stopped smoking during the first 10 weeks, 1 showed a significant increase in FeNO two weeks later, which therefore may have been a false positive induced by tobacco stop. Among the 11 patients who developed respiratory symptoms without increase in FeNO, 1 received corticosteroids between 6 and 10 weeks, which may have at least partially blunted NO increase and lead to a false negative.

## Discussion

The main findings of this study are the following. In lung cancer, small increases in FeNO (4 ppb, 27% of baseline value) and of alveolar NO were observed 7.5 months after the beginning of RT. Thirteen (20%) lung cancer patients showed significant increases (>10 ppb) in FeNO within the 10 weeks after RT beginning. The specificity of FeNO increases > 10 ppb for predicting radiation-induced symptoms was high (83%), but the sensitivity was very low (18%). Eventually, there was no association between changes in FeNO and CT scan images or with DVH parameters in the lung cancer group.

The short-term impact of chemo and radiotherapy on FeNO has been described in a few studies. Before treatment, FeNO of lung cancer patients has been found to be ~2.5 fold that of controls [8,9]. Eight days after carboplatin or cisplatin-based chemotherapy, FeNO decreased by 3.8 ppb in 39 lung cancer patients and returned to baseline values at days 15 [10]. After RT or combined chemo-RT, in 29 patients, FeNO decreased by 35% at 40 Gy and just after completion of RT [11]. In our study, baseline FeNO was in the range of normal values, but was measured after chemotherapy in almost all lung cancer patients. Chemotherapy may have “normalized” FeNO values. RT did not reduce FeNO in our patients. It is well known that a tumour and its microenvironment produce NO, which may promote tumour growth mainly by stimulating angiogenesis. In lung cancer, the main source of exhaled NO has been attributed to lung macrophages [8,10]. Indeed, the magnitude of the inducible nitric oxide synthase (iNOS) expression in alveolar macrophages has been found to correlate with the level of exhaled NO, and immunohistochemical studies indicated that alveolar macrophages is likely the major cellular source of NO production [8]. In another study, the decrease in FeNO observed at day 8 after chemotherapy paralleled the decrease in monocytes in peripheral blood [10]. The decrease in FeNO observed after treatment of these chest malignancies is believed to be due to cell death in the tumour and its microenvironment and/or reduction of tumour-associated inflammation.

To our knowledge, this study is the first to provide measurements of FeNO several months after RT for chest malignancies. In lung cancer patients, FeNO showed a small increase 4 and 7.5 months after the beginning of radiotherapy, likely due to an increase in alveolar NO. The origin of this increase may be the recurrence or progression of the lung tumour. Tumour recurrence or progression was diagnosed in 17 lung cancer patients in our series, but cannot be totally excluded in other patients, since this diagnosis when based on CT-scan imaging may be difficult after RT. Another explanation may be the development of chemotherapy- or radiation-induced lung damages. Indeed, if low-intermediate chronic NO doses may promote tumour development/progression [22], radiation therapy may produce high levels of NO, which exert cytotoxic effects. These cytotoxic effects may have antitumor properties, but may also induce damages in normal tissues. In mice, alveolar macrophages produced NO after irradiation, and expression of iNOS in both alveolar macrophages and alveolar epithelial cells are increased. Moreover, the progression of radiation pneumonitis could be reduced with NOS inhibitor treatment [23].

Three human studies assessed the predictive value of FeNO for radiation pneumonitis symptoms. The first one included 28 oesophagal cancer patients and found that a 1.5-fold increase in FeNO had a 100% sensitivity and 100% specificity [14]. In another study by the same team, the ratio of FeNO at the end of RT to pre-RT was calculated in 50 lung or oesophageal cancer patients. A threshold of 1.4 perfectly separated symptomatic and asymptomatic patients [15]. In the third study, 5 lung cancer patients (17%) demonstrated a three-fold increase in FeNO during RT. Three out of these 5 patients had radiation pneumonitis requiring steroids [11]. In our study, the specificity of a 10 ppb increase of FeNO for detecting radiation pneumonitis symptoms in lung cancer patients was quite good (83%), but the sensitivity was low (18%). Using a lower threshold (increase >4 ppb, the lowest significant increase with our device) did not improve sufficiently the sensitivity of the test, which reached only 36%. Eventually, using a >2 fold increase (associated with a minimal, >4 ppb increase) as cut point did not allow a better detection of radiation-induced symptoms compared to the 10 ppb cut-off value.

The low sensitivity of FeNO to detect radiation pneumonitis may be due to the development of respiratory symptoms from other causes than radiation pneumonitis, such as exacerbation of underlying lung disease and respiratory infections, or to the difficulty to evaluate accurately dyspnea variations for patients with pre-existing symptoms [24]. This is of real concern in case of populations characterized by a high proportion of patients with pre-treatment pulmonary disease (75% of our patients had impaired pre-treatment lung function) [24]. However, interestingly, the sensitivity of FeNO in our study did not increase dramatically (18% to 22%) when the diagnosis of radiation lung injury was based on either symptoms and/or CT scan images. Another reason for the low sensitivity of FeNO may be the use of measurements performed at a unique flow rate of 50 ml/s (FeNO_50_). At this flow rate, NO, if not extremely elevated, is mainly of bronchial origin. In scleroderma, alveolar concentration of NO, but not FeNO_50_, predicted degradation of lung function [3]. Given that we showed alveolar NO varies with time after RT, follow-up of the alveolar fraction of exhaled NO might be more accurate than that of FeNO_50_ to detect radiation pneumonitis, and deserves to be studied in larger populations. Eventually, the response of the lung to radiation may vary from a patient to another, with activation of various inflammatory pathways, and diverse patterns of NO production. Interestingly, a sporadic form of radiation pneumonitis has been described, characterized by a bilateral lymphocytic alveolitis, and similar to hypersensitivity pneumonitis [25]. It would be of interest to analyse the bronchoalveolar lavage from patients with very strong and sustained increases in FeNO, such as the three patients described in this study, looking for lymphocytic alveolitis.

Our study was conducted in patients treated with RT, whatever the lung cancer treatment prescribed prior to or concomitant with RT. This heterogeneity of patients may explain partly the low sensitivity of FeNO to predict radiation-induced pneumonitis, since some chemotherapy agents may have modulated the lung radiation-induced toxicity and/or NO production. However, today, lung cancer treatments frequently combine various chemotherapy agents and RT modalities, and the interest of FeNO measurements in the daily practice would be limited if restricted to the follow-up of a particular subgroup of patients. Moreover, numerous other factors, such as corticosteroids, smoking, diet, may also have confounding effects and add difficulty in interpreting FeNO variations in these patients. In our study, tobacco smoking and corticosteroid treatments were taken into account and their potential effects could barely account alone for the low sensitivity of FeNO. Eventually, false positive are also a real issue. In our department, 2 out of 20 Hodgkin lymphoma patients without radiation pneumonitis showed unexplained, >10 ppb increases in FeNO after RT (Enache I, unpublished data).

To conclude, in this study, a small proportion of lung cancer patients developed symptoms of radiation pneumonitis, and serial FeNO measurements during RT had a low ability to identify them. A larger proportion of patients developed radiation pneumonitis images, but FeNO variations did not correlate with the score of these images. Three patients developed both large and sustained increases in FeNO together with significant radiation pneumonitis symptoms or images, which might reflect a particular lung response to radiation.

## Competing interest

The authors declare that they have no competing interests.

## Authors’ contribution

IE and EQ participated in the study design and coordination and helped to draft the manuscript. AC conceived of the study, and participated in its design and drafted the manuscript. GN and CS participated in the acquisition and interpretation of the radiotherapy data. MOM, EUK and BG participated in the acquisition and interpretation of pulmonary function data (including FeNO measurements). MYJ analyzed all the CT-scan. NM performed the statistical analysis. All authors read and approved the final manuscript.
